# Proteomics of Gnathostomiasis: A Way Forward for Diagnosis and Treatment Development

**DOI:** 10.3390/pathogens10091080

**Published:** 2021-08-25

**Authors:** Tipparat Thiangtrongjit, Kathyleen Nogrado, Thawatchai Ketboonlue, Preeyarat Malaitong, Poom Adisakwattana, Onrapak Reamtong

**Affiliations:** 1 Department of Molecular Tropical Medicine and Genetics, Faculty of Tropical Medicine, Mahidol University, Bangkok 10400, Thailand; tipparat.thi@mahidol.ac.th (T.T.); ksnogrado@gmail.com (K.N.); 2 Department of Helminthology, Faculty of Tropical Medicine, Mahidol University, Bangkok 10400, Thailand; thawatchai.ket@mahidol.edu (T.K.); preeyarat.mal@mahidol.ac.th (P.M.)

**Keywords:** gnathostomiasis, *Gnathostoma spinigerum*, proteomics, diagnosis, treatment

## Abstract

*Gnathostoma spinigerum* is the most common cause of gnathostomiasis in humans. It has a complex life cycle, which requires two intermediate hosts and a definitive host, and poses a high risk for zoonosis. Definitive prognosis of gnathostomiasis relies mainly on the isolation of advanced-stage larvae (aL3), which is very challenging especially if the aL3 is sequestered in difficult-to-reach organs. There is also a lack of a confirmatory diagnostic test for gnathostomiasis. With the ongoing advancement of proteomics, a potential diagnostic approach is underway using immunoproteomics and immunodiagnostics. In addition to this, the employment of mass spectrometry could further elucidate not only understanding the biology of the parasite but also determining potential targets of prospective drugs and vaccines. This article reports the past, present, and future application of proteomics in the study of gnathostomiasis.

## 1. Biology and Distribution of Gnathostoma

**Figure 1:**
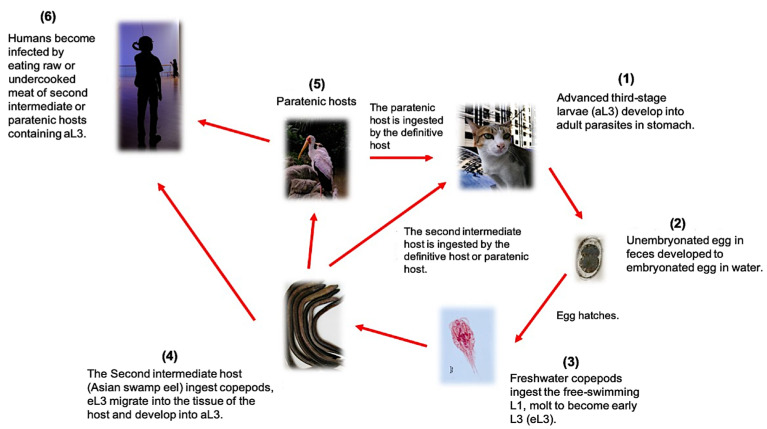
The complicated life cycle of *G. spinigenum* illustrating the definitive and paratenic hosts.

**Figure 2:**
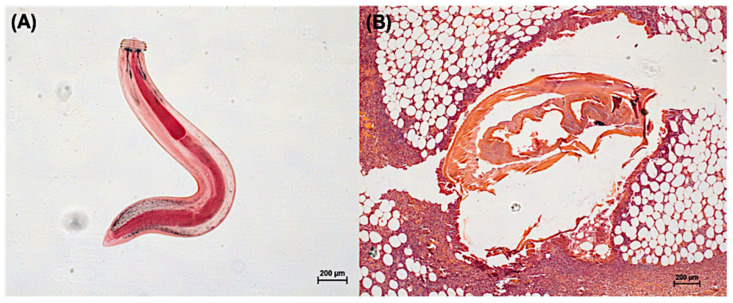
Photo indicating the aL3 obtained from the Asian swamp eels (*Monopterus albus*) (**A**) and a cross-sectional picture of a tissue biopsy of a patient with mastitis showing the aL3 causing cutaneous larva migrants (**B**). All photos were generated in our laboratory to demonstrate *G. spinigenum* aL3 morphology.

The genus *Gnathostoma* belongs to the order Spirurida and is one of the groups of parasitic nematodes that prevail in the tropical and subtropical regions. Human gnathostomiasis is endemic in Southeast Asia (Thailand, Laos, Myanmar, Indonesia, Malaysia, and the Philippines) and Japan, where people eat raw freshwater fish or shellfish [1]. However, it is now considered an emerging infectious disease due to increasing occurrence in non-endemic areas such as Central and South America particularly Mexico [2]. The definitive hosts of this parasite are wild and domestic cats, dogs, pigs, rats, and weasels. Currently, a total of 18 species have been described belonging to this genus, but only six species of these are known to infect humans, namely *G. binucleatum*, *G. doloresi*, *G. bispidum*, *G. malaysiae*, *G. nipponicum*, and *G. spinigerum*, which are geographically distributed in Asia, Central and South America, Australia, and East Africa [3,4]. Presently, research is needed to improve the diagnosis of gnathostomiasis. Among the previously mentioned human-infecting species of *Gnathostoma*, *G. spinigerum* has the most complicated life cycle and poses a higher risk for zoonotic transmission. Thus, it will be given the emphasis in this review [5]. *G. spinigerum* requires two intermediate hosts and one definitive host to complete its life cycle (Figure 1). In general, the adult worms live in the wall of the stomach of the definitive host (carnivores like canines and felids) and lay eggs, which are then released into the environment through the feces. The first-stage larvae (L1) hatch from the eggs in the freshwater and will be ingested by the first intermediate hosts, copepods (usually cyclops species), in which they will develop into second-stage larvae (L2) and early third-stage larvae (eL3). When the second intermediate hosts or paratenic hosts such as fishes, frogs, and snakes ingest the cyclops spp., the eL3 will migrate into the tissue and then encyst and develop into the aL3 (Figure 2A). In the case that other paratenic hosts, for example, reptiles and birds, ingest the secondary intermediate host containing aL3, the larvae will not develop further. The life cycle will only be completed when the definitive hosts (cat or dog) ingest the second intermediate or the paratenic host harboring the aL3. The aL3 could migrate to the stomach and form the tumor-like mass at the stomach wall to mature, mate, and lay eggs. It is important to note that humans are accidental hosts, as the larvae cannot develop in humans and remain as aL3. However, aL3 can migrate to skin or visceral organs, causing severe clinical manifestations [4].

## 2. Gnathostomiasis as Public Health and Travel Medicine Problem

Gnathostomiasis is a parasitic disease caused by a nematode of the genus *Gnathostoma*. Several reports of the disease have been recorded in Asia and Central America [6]. There are sporadic cases in non-endemic countries when travelers return home after visiting the endemic areas, especially Southeast Asia [2,7]. *G. spinigerum* is the major species causing human gnathostomiasis in Southeast Asia, particularly in Thailand. Subcutaneous or cutaneous intermittent migratory swelling with peripheral eosinophils are the clinical manifestations frequently observed during parasitic infection. However, severe cases can potentially occur when the parasite migrates to visceral organs such as the brain, spinal cord, and eyes [8,9,10]. Clinical signs and symptoms, in addition to a history of ingestion of raw or improperly cooked second intermediate hosts or paratenic hosts of *Gnathostoma* in the endemic areas, are useful information for proper diagnosis [11]. Alternatively, immunodiagnosis has been developed and used as a supportive diagnostic method for gnathostomiasis [12].

## 3. Clinical Manifestations of *Gnathostoma* Larva Migration

The clinical manifestations of gnathostomiasis can be categorized into cutaneous and visceral forms. Cutaneous gnathostomiasis is the most common manifestation. Intermittent migratory swellings usually affect the trunk or upper limbs, with non-pitting edematous type that varies in size and can be pruritic, painful, or erythematous (Figure 2B). The symptoms can show within 3 to 4 weeks after ingestion of the larvae [13,14]. The swellings are due to both mechanical damages caused by the larvae and the host’s immunological response to the parasite and its secretions. Regrading larval migration, subcutaneous hemorrhage may be observed along its track specific to gnathostomiasis. It can help differentiate it from other causes of larva migrants such as sparganosis, hookworm infection, or strongyloidiasis [1].

In comparison to the cutaneous type, the visceral form is more harmful. This occurs when the larvae penetrate deep into the internal organs. The severity and symptoms of the disease vary according to the invaded target organs. If the larvae invade the lung, the symptoms are pleuritic chest pain, hemoptysis, lobar consolidation, collapse, pleural effusions, and pneumo- or hydropneumothorax [15,16,17]. On the other hand, when the larvae invade the eye, uveitis intraocular hemorrhages, glaucoma, retinal scarring, and detachment may be observed [18]. In the most severe case, when the brain and spinal cord are breached, it may cause limb weakness, paralysis, unconsciousness, and death [10,19]. 

## 4. Treatment, Prevention, and Diagnosis of Gnathostomiasis

The recommended treatments rely on surgical removal or treatment with albendazole or ivermectin. Although the best treatment for gnathostomiasis is the surgical removal of the larva, this is only effective when worms are located in an accessible location. For surgery, medications such as albendazole and ivermectin have also been noted for their efficacy in eliminating the parasite [20]. Administration of albendazole at 400 mg/day for 21 days is recommended, with cure rates between 93.9% and 94.1%. Unfortunately, this regimen elicits adverse side effects such as gastrointestinal distress, headache, dizziness, increasing and reversible levels of hepatic enzymes, and transient reduction of the total leukocyte count [21]. On the other hand, treatment with a single dose (150–200 μg/kg) of ivermectin showed a high curability of cutaneous larva migrants (creeping eruption) at a 100% cure rate without significant adverse effects [22]. However, a previous clinical trial suggested that a single dose of ivermectin (200 μg/kg) was less effective than albendazole (400 mg/day for 21 days) for treatment of cutaneous gnathostomiasis [23].

Avoiding the consumption of raw or undercooked meat is essential in the prevention of gnathostomiasis. However, changing the eating habits of people in endemic areas is difficult. In endemic areas such as Southeast Asia, there are traditional dishes that use raw or undercooked fish such as koipla in Thailand, goi ca song in Vietnam, and sashimi and sushi in Japan [24]. Health education regarding these traditional eating behaviors needs to be emphasized. The most important methods of their food preparation in order to kill the larvae without greatly altering the taste of traditional dishes should be practiced. For instance, meat should be marinated in vinegar for 6 h or in soy sauce for 12 h to kill the larvae successfully. In areas with reliable electricity, meat can be frozen at −20 °C for 3 to 5 days to achieve the same results of getting rid of the larvae [25].

Definite diagnosis for gnathostomiasis is challenging. Firstly, clinical manifestations and history of eating raw foods are used to measure for suspected gnathostomiasis; however, these are still indistinguishable from the signs and symptoms of angiostrongyliasis, trichinellosis, and hookworm cutaneous larva migrants [1]. Secondly, eosinophilia in the cerebrospinal fluid (CSF) is distinct evidence for the diagnosis of gnathostomiasis. The observed level of eosinophil is found at 5 to 94% with a high total CSF white cell count up to an average of 500 cells/mm^3^ (20–1420 cells mm^3^) [26]. However, this eosinophilia is still a non-specific issue as it has also been found to occur when infected with several other parasites such as *Angiostrongylus cantonensis*, *Toxocara canis*, *Strongyloides stercoralis*, *Ascaris lumbricoides*, *Paragonimus westermani*, *Fasciola hepatica*, *Trichinella spiralis*, schistosomes, and other infections such as coccidiodomycosis and *Aspergillus* infection [1,10]. Alternatively, immunodiagnosis has been developed over the years and is promising a high sensitivity and specificity that may be advantageous in the differential diagnosis of gnathostomiasis [27].

## 5. Immunodiagnosis Is Appropriate Method for Diagnosis of Gnathostomiasis

Antigen-based diagnosis by capturing circulating antigen in serum is an ideal method for human gnathostomiasis. However, low sensitivity was reported according to the lack of an adequate amount of antigen presented in a clinical specimen. Antigen-capture sandwich enzyme-linked immunosorbent assay (ELISA) of CSF from 11 suspected patients found that only three of them gave positive results and only one case was positive with parasitological confirmation [3]. Afterward, antibody-based diagnoses for detection of gnathostomiasis was developed and showed higher power in terms of sensitivity over antigen test. ELISA based on the detection of human IgG class antibody (total IgG) to *Gnathostoma* antigen was widely used in the past few decades [28,29,30,31]. Recently, Western blot analysis to detect specific total IgG against 24 kDa protein in *G. spinigerum* third-stage larva (L3) extract has been used as a standard diagnosis [32]. Not only total IgG but also detection of specific IgG subclasses against 24 kDa antigen was performed. The result showed that IgG4 gave the best sensitivity and specificity at 91.6% and 87.8%, respectively, comparing among other subclasses [33]. Although detection of specific IgG against crude worm antigen (CWA) elicits high sensitivity and specificity, the whole process of antigen preparation is quite complicated, time-consumable and laborious, and non-quality batch-to-batch. Furthermore, the collection of *G. spinigerum* L3 from natural sources is limited depending on season and environmental conditions. To improve antigen preparation, recombinant protein technology has been applied to produce mimic antigens for further development of reliable diagnosis. However, the sensitivity and specificity of recombinant protein compared to a crude antigen is still doubtful and needs to be validated further [12,34,35].

The mRNA encoding the 24 kDa protein was first identified as matrix metalloprotease (GsMMP) by immunoscreening with the monoclonal antibody (mAb GN6) [36]. Recombinant GsMMP (rGsMMP) expressed by prokaryotic expression systems could effectively diagnose neurognathostomiasis with high sensitivity (100%) and specificity (100%) [37]. However, validation of rGsMMP with several other related diseases still needs to confirm the accomplishment. Cathepsin L (GsCL1) was identified from the λZAP cDNA library of *G. spinigerum* aL3, and the recombinant protein (rGsCatL) was produced and successfully elicited enzymatic activity [38]. In our previous study, rGsCatL expressed by the prokaryotic system exhibited high cross-reaction with other heterologous infected sera (unpublished personal data). However, eukaryotic expression systems such as yeast, mammalian cell, or insect cell may be adapted for correct conformation or modification of the rGsCatL. [38,39]. Cyclophilin (CyP) has been identified from *G. spinigerum* aL3 and recognized by human gnathostomiasis sera using two-dimensional electrophoresis (2-DE) and liquid chromatography in conjunction with tandem mass spectrometry (LC-MS/MS) [40]. Recombinant GsCyP (rGsCyP) used for immunoblotting solely reacted with human gnathostomiasis sera but not with healthy control or other parasite-infected sera [12]. However, a sufficient number of sera with various diseases are still required to ensure diagnostic efficacy. Until now, only a few antigens have been employed as candidates for recombinant protein-based immunodiagnosis. Therefore, new immunodiagnosis candidates must be investigated further. Proteomics is a powerful discovery tool to identify candidate immunodiagnostic antigens in several pathogens, including *G. spinigerum*.

## 6. Proteomics for Identifying Immunodiagnostic Candidates and Drug Targets of *G. spinigerum*

Proteomics is the large-scale study of proteins in complex biological samples. A workflow basically consists of protein digestion, liquid chromatography (LC) separation, mass spectrometry (MS), and data interpretation [41]. Proteomic approaches can be used for proteome profiling, comparative quantification, localization, posttranslational modifications, and protein–protein interactions. Recently, proteomics has been applied to investigate parasite proteomes, which are essential to understand disease pathobiology and design novel interventions [42]. Although applying this technology to other helminth parasites has been reported [43], only a few publications were done on *Gnathostoma.* Immunoproteomics and secretome are currently the only two types of proteomics that have been done on *Gnathostoma*.

Parasitic helminths producing excretory–secretory proteins (ESPs) are required for food intake, tissue penetration, and host–parasite interactions [44]. Moreover, studies on ESPs could be useful for diagnostic biomarker discovery, as these proteins are released from the parasites and circulate in the host cell environment [45]. The ESPs represent a complex mixture of molecules that have been transported via secretory pathways and sloughed off the tegument. ESPs suppress the host immune system and aid parasite survival [46]. Therefore, the secretome data could be useful for therapeutic target identification, diagnostic tools, and pathogen control. MS-based proteomics coupled with the in-house cDNA-transcribed library were used to reveal *G. spinigerum* aL3 secretome [40]. This research identified 29 *G. spinigerum* ESPs. Many proteins are involved in signaling transduction, transcriptional control, transportation, and programmed cell death. A metalloendopeptidase and a serine carboxypeptidase were discovered as predominant proteases in the *G. spinigerum* secreted product. Metalloproteases released by *G. binucleatum* aL3 were found to degrade gelatin, fibronectin, and antibodies in a prior study, suggesting that they may play a role in *Gnathostoma* tissue invasion, migration, and immune evasion [47]. For that reason, metalloprotease inhibitors may reduce collateral tissue damage of gnathostomiasis. Interestingly, doxycycline is clinically approved for therapy of severe gum infection on account of the inhibition of collagenases rather than antibacterial effects [48]. Thus, the use of antibiotics as a metalloprotease inhibition might be a complementary therapeutic for parasitic infection. Serpin was also identified in *G. spinigerum* ESPs. Serpins are mostly inhibitors of serine proteases. Non-inhibitory actions of serpins including molecular chaperone activity, hormone transfer, and tumor suppression have been reported. The parasites are protected from the host proteolysis by the secretion of serpins, which also aids the worms in invading the host-defensive barriers and evading the host immunological response [49]. Vaccination of mice with *T. spiralis* serpin exhibited 62.2% and 57.25% reduction in intestinal adult worm and muscle larvae, respectively [50]. The *G. spinigerum* serpin might also be a potential vaccine and drug target against gnathostomiasis. The functions of other 19 *G. spinigerum* ESPs are still unknown, and more characterizations are required for understanding their roles and functions.

**Figure 3:**
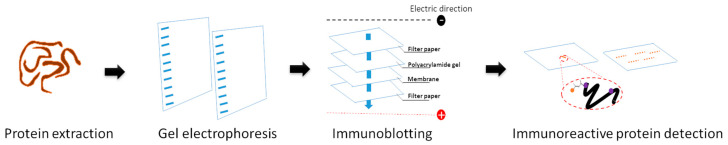
A diagram illustrating the general immunoproteomic workflow.

Another proteomics study on *Gnathostoma* was the profiling of *Gnathostoma* antigens using immunoproteomics. The immunoproteomics is a technique that combines protein separation, immunological detection, and MS analysis to reveal antigens that induce host immune responses. In this method, 2-DE is used to separate proteins from cells or tissues. Each gel is electro-transferred onto a nitrocellulose membrane, and immunoblotting is performed with the infected host serum. The infected serum contains antibodies that can recognize parasite antigenic proteins. The antigen–antibody reaction is visualized by enzyme-labeled secondary antibodies. Afterward, the antigenic spots are excised and analyzed using LC-MS/MS. The immunoproteomics workflow is demonstrated in Figure 3 [51]. This technique facilitates the search for candidate antigens leading to diagnostics and vaccine development, as well as providing a better understanding of the host–parasite interaction. *G. binucleatum* is the causative parasite of human gnathostomiasis in the Americas. *G. binucleatum* immunodominant antigens generated from somatic antigens and excretory–secretory antigens have been studied by two-dimensional immunoblot analysis. Sera were collected from 16 patients with gnathostomiasis, which were diagnosed either by recovery of larva or by a positive ELISA result. The metazoa subset of the NCBInr database was used for protein identification. Two spots of CWA (32 kDa; pI 6.3 and 6.5) were identified as type 1 galectins by mass spectrometric analysis [52]. According to *G. spinigerum*, two studies focused on CWA, and one study focused on ESPs. Immunoproteomics was used to identify the reactive spots of *G. spinigerum* aL3 CWA. Six parasitologically confirmed cases of human gnathostomiasis served as positive control sera. While a negative control serum was created by pooling the sera of 30 healthy Thai individuals with no history of infection and migrating cutaneous swellings and who were free of parasite infections in the intestine. Proteins with a molecular weight of 23 to 24 kDa and a pI of 8.1 to 9.3 were recognized by human gnathostomiasis serum. The nr.fasta database was used to identify proteins in these two gel plugs. Cyclophilin, hypothetical protein, actin, matrix metalloproteinase-like protein, and intermediate filament protein B were identified as candidates for gnathostomiasis diagnosis [53]. A more recent study on immunoproteomic analysis of CWA of *G. spinigerum* aL3 was reported. Pooled positive serum was gathered from thirteen confirmed cases of adult human gnathostomiasis. At the same time, thirty healthy adult volunteers provided pooled negative reference serum. The 93 antigenic spots were excised, and protein identification was accomplished by searching against the NCBI protein database (all entries). Twenty-seven proteins could be identified by LC-MS/MS [54]. The immunoproteomics analysis was applied not only to *G. spinigerum* aL3 somatic proteins but also to aL3Gs-ESPs. Protein identification was performed against an in-house transcriptome database, and 15 proteins could be identified [40]. Summary of important *Gnathostoma* antigens identified by immunoproteomics studies is presented in Table 1. Immunoproteomics successfully explored *Gnathostoma* immunogen datasets. However, the candidates need to be further validated as diagnostic and vaccine candidates. The sensitivity and specificity data are required for gnathostomiasis diagnosis. The estimation of vaccines’ protective efficacy is necessary for further development. Moreover, the large-scale protein production and evaluation of the capacity of those proteins should be investigated further.

## 7. Future Perspectives of Proteomics in Improving Diagnosis and Treatment of Gnathostomiasis

Immunoproteomics is successfully applied for *G. spinigerum* immunogen identification. However, antigen profiles of *G. spinigerum* proteins, which could be recognized by other host immune systems such as dog, cat, and cattle remain unknown. This information is also useful for the prevention and control of gnathostomiasis since there is also a lack of diagnosis and vaccine in different hosts. Moreover, other types of proteomic studies are limited to *Gnathostoma*. To perform a proteomic analysis, an adequate amount of biological sample is required. *Gnathostoma* nematodes require two intermediate hosts and one definitive host to complete their life cycle. Freshwater copepods are the first intermediate host, while fish or tadpoles are the second intermediate host. A dog or a cat is the definitive host. The life cycle of *G. spinigerum* is difficult to sustain in the laboratory since the parasite hosts are not common laboratory animals. As a result, a sufficient volume of material must be prepared from natural infection. To obtain the aL3Gs, livers of naturally infected eels are extracted using an acid-pepsin digestion process. This procedure is dependent on the presence of eels, and only one stage can be collected [54,55]. As the *G. spinigerum* develops through numerous stages, the profiles of protein expression change dramatically. An ideal novel anthelminthic drug should target a protein present in all life-cycle stages of the *G. spinigerum* to have the greatest treatment efficiency. Gaining the information of stage-specific pathways is important for underlining the essential proteins involved in parasite adaptation to its human host. Inhibition of parasite stage development process is one approach for antiparasitic drug design. However, the complete set of proteins expressed in all *G. spinigerum* stages are unavailable. Another obstacle of global proteomic analysis is the lack of a genome database for *G. spinigerum*. This limitation will result in incomplete proteomics identification. According to our unpublished results, using our in-house *G. spinigerum* transcriptome database will yield substantially more protein identification than using the public database. Therefore, it is also critical to investigate the entire genome of *G. spinigerum*. The proteomic analysis could also be used to study post-translation modifications (PTMS), for example, glycosylation and phosphorylation. Glycosylation on the surface proteins and ESPs of parasites play essential roles in the ability of adaptation and survival in different hosts and environments. Since the glycan of parasites could modulate host immune responses, these molecules are important for diagnostics and vaccinology [56]. Protein phosphorylation is eukaryotic cells’ major regulatory mechanism for controlling cellular functions. Understanding the relationship between protein kinases, phospho-signaling cascades, and protein substrates is critical for understanding the mechanisms that regulate cellular activity as well as identifying potential therapeutic targets. Identification of *G. spinigerum* PTMs might become increasingly relevant candidates for the diagnosis, vaccine, and drug development of gnathostomiasis [57,58,59,60].

## Figures and Tables

**Table 1:** Summary of important *Gnathostoma* antigens identified by immunoproteomics studies.

Antigen	Separation-	Database for Protein Identification	Protein Identification	Ref.
*G. binucleatum* CWA	pH 5–8, 12% gel	Metazoa of NCBInr	Galectins	[52]
*G. binucleatum* ESP	pH 5–8, 12% gel	Metazoa of NCBInr	-	[52]
*G. spinigerum* CWA	pH 3–11, 12% gel	nr.fasta database	Cyclophilin Hypothetical protein Actin Matrix metalloproteinase-like Intermediate filament protein B	[53]
*G. spinigerum* CWA	pH 3–11, 12% gel	All entries of NCBI protein database	As37 Actin 2 Heat shock protein 90 Heat shock protein 70 Chaperonine protein HSP60 Chaperone protein DnaK Phosphoenolpyruvate Carboxykinase domain-containing Protein Carboxyl transferase domain protein Enolase Glyceraldehyde-3-phosphate dehydrogenase Peptidyl-prolylcis-trans isomerase Cyclophilin Cytoplasmic intermediate filament protein Peroxiredoxin Matrix metalloproteinase-like protein Fructose-bisphosphate aldolase 53 kDa Excretory/secretory protein Glu/Leu/Phe/Val dehydrogenase, dimerization domain protein Galectin methylmalonyl 4-Hydroxybutyrate coenzyme a transferase Phosphoglycerate mutase Eukaryotic translation elongation factor 1A Phosphoglycerate kinase Proteasome subunit alpha type 7-1 Myosin heavy chain Cytoplasmic Cu/Zn-superoxide dismutase CBN-MCE-1 protein ATPase and cell division protein 48 and Vps4 oligomer ization Kinesin-2	[54]
*G. spinigerum* ESP	pH 3–10, 12% gel	In-house transcriptome	Serine_rich_NEDD9 Catalase heme-binding enzyme Pyrroline-5-carboxylate reductase Phospho-2-dehydro-3-deoxyheptonate aldolase Serpin AJAP1/PANP C-terminus	[40]

## Data Availability

Not applicable.

## References

[1] Herman J.S., Chiodini P.L. (2009). Gnathostomiasis, another emerging imported disease. Clin. Microbiol. Rev..

[2] Moore D.A., McCroddan J., Dekumyoy P., Chiodni P.L. (2003). Gnathostomiasis: An emerging imported disease. Emerg. Infect. Dis..

[3] Chaicumpa W. (2010). Immunodiagnosis of gnathostomiasis. Siriraj Med. J..

[4] Diaz J.H. (2015). Gnathostomiasis: An emerging infection of raw fish consumers in *Gnathostoma* nematode-endemic and nonendemic countries. J. Travel Med..

[5] Ontranto D., Deplazes P. (2019). Zoonotic nematodes of wild carnivores. Int. J. Parasitol. Parasites Wildl..

[6] Almeyda-Artigas R.J., Bargues M.D., Mas-Coma S. (2000). ITS-2rDNA Sequencing of *Gnathostoma* species (Nematoda) and elucidation of the species causing human gnathostomiasis in the Americas. J. Parasitol..

[7] Katchanov J., Sawanyawisuth K., Chotmongkol V., Nawa Y. (2011). Neurognathostomiasis, a neglected parasitosis of the central nervous system. Emerg. Infect. Dis..

[8] Visudhipan P., Chiemchanya S., Somburanasin R., Dheandhano D. (1980). Causes of spontaneous subarachnoid hemorrhage in Thai infants and children. A study of 56 patients. J. Neurosurg..

[9] Chitanondh H., Rosen L. (1967). Fatal Eosinophilic Encephalomyelitis Caused by the Nematode Gnathostoma Spinigerum. Am. J. Trop. Med. Hyg..

[10] Boongird P., Phuapradit P., Siridej N., Chirachariyavej T., Chuachirun S., Vejjajiva A. (1977). Neurological manifestations of gnathostomiasis. J. Neurol. Sci..

[11] Nopparatana C., Chaicumpa W., Tapchaisri P., Setasuban P., Ruangkunaporn Y. (1992). Towards a suitable antigen for diagnosis of Gnathostoma spinigerum infection. Int. J. Parasitol..

[12] Laummaunwai P., Intapan P.M., Wongkham C., Lulitanond V., Tayapiwatana C., Maleewong W. (2010). Gnathostoma spinigerum: Molecular cloning, expression and characterization of the cyclophilin protein. Exp. Parasitol..

[13] Daengsyang S. (1949). Human gnathostomiasis in Siam with reference to the method of prevention. J. Parasitol..

[14] Rusnak J.M., Lucey D.R. (1993). Clinical Gnathostomiasis: Case Report and Review of the English-Language Literature. Clin. Infect. Dis..

[15] Dow C., Chiodini P., Haines A., Michelson S. (1988). Human gnathostomiasis. J. Infect..

[16] Nagler A., Pollack S., Hassoun G., Kerner H., Barzilai D., Lengy J. (1983). Human pleuropulmonary gnathosomiasis: A case report from Israel. Isr. J. Med. Sci..

[17] Parola P., Bordman G., Brouqui P., Delmont J. (2004). Eosinopilic pleural effusion in gnathostomiasis. Emerg. Infect. Dis..

[18] Biswas J., Gopal L., Sharma T., Badrinath S.S. (1994). Intraocular *Gntathostoma spinigerum*. Clinicopathologic study of two cases with review of literature. Retina.

[19] Punyagupta S., Bunnag T., Juttijudata P. (1990). Eosinophilic meningitis in Thailand. Clinical and epidemiological characteristics of 162 patients with myeloencaphalitis probably caused by *Gnathostoma spinigerum*. J. Neurol. Sci..

[20] Nontasut P., Bussaratid V., Chullawichit S., Charoensook N., Visetsuk K. (2006). Comparison of ivermectin abd Albendazole treatment for Gnathostomiasis. Southeast Asian J. Trop. Med. Public Health.

[21] Kraivichian K., Yentakam S., Nuchprayoon S., Sitichalernchai P., Chaicumpa W. (2004). Treatment of cutaneous gnathostomiasis with ivermectin. Am. J. Trop. Med. Hyg..

[22] Kraivichian P., Kulkumthorn M., Yingyourd P., Akarabovorn P., Paireepai C.-C. (1992). Albendazole for the treatment of human gnathostomiasis. Trans. R. Soc. Trop. Med. Hyg..

[23] Caumes E., Carriere J., Datry A., Gaxotte P., Danis M., Gentilini M. (1993). A randomized trial of ivermectin versus albendazole for the treatment of cutaneous larva migrants. Am. J. Trop. Med. Hyg..

[24] Sorvillo F.J. (2008). Food-borne parasitic zoonoses: Fish and plant-borne parasites (World class parasites). Emerg. Infect. Dis..

[25] Hale D.C., Blumberg L., Frean J. (2003). Case report: Gnathostomiasis in two travelers to Zambia. Am. J. Trop. Med. Hyg..

[26] Wang C.H., Gao S.F., Guo Y.P. (1993). Diagnostic significance of eosinophilia of the cerebrospinal fluid in cerebral cysticercosis. Chin. Med. J..

[27] Zambrano-Zaragoza J.F., Durán-Avelar M.D.J., Vibanco-Pérez N., Messina-Robles M. (2012). Characterization of the Humoral Immune Response against Gnathostoma binucleatum in Patients Clinically Diagnosed with Gnathostomiasis. Am. J. Trop. Med. Hyg..

[28] Diaz-Camacho S.P., Zazueta-Ramos M., Ponce-Torrecillas E., Osuna-Ramirez I., Castro-Velasquez R., Flores-Gaxiola A., Baquera-Heredia J., Willms K., Akahane H., Ogata K. (1998). Clinical manifestations and immunodiagnosis of gnathostomiasis in Culiacan, Mexico. Am. J. Trop. Med. Hyg..

[29] Suntharasami P., Desakron V., Migasena S., Bunnag D., Harinasuta T. (1985). ELISA for immunodiagnosis of human gnathosomiasis. Southeast Asian J. Trop. Med. Public Health.

[30] Dharmkrong-At A., Mmigasena S., Suntharasamai P., Bunnag D., Priwan R., Sirisinha S. (1986). Enzyme-Linked Immunosorbent Assay for detection of antibody to *Gnathostoma* antigen in patients with intermittent cutaneous migratory swelling. J. Clin. Microbiol..

[31] Maleewong W., Morakote N., Thamasonthi W., Charuchinda K., Tesana S., Khamboonruang C. (1988). Serodiagnosis of human gnathostomiasis. Southeast Asian J. Trop. Med. Public Health.

[32] Tapchaisri P., Nopparatana C., Chaicumpa W., Setasuban P. (1991). Specific antigen of *Gnathostoma spinigerum* for immunodiagnosis of human gnathostomiasis. Int. J. Parasitol..

[33] Laummaunwai P., Sawanyawisuth K., Intapan P.M., Chotmongkol V., Wongkham C., Maleewong W. (2007). Evaluation of human IgG class and subclass antibodies to a 24 kDA antigenic component of *Gnathostoma spinigerum* for the serodiagnosis of gnathostomiasis. Parasitol. Res..

[34] Saenseeha S., Penchom J., Yamasaki H., Laummaunwai P., Tayapiwatana C., Kitkhuandee A., Maleewong W., Intapan P.M. (2014). A dot-ELISA test using a *Gnathostoma spinigerum* recombinant matrix metalloproteinase protein for the serodiagnosis of human gnathostomiasis. Southeast Asian J. Trop. Med. Public.

[35] Janwan P., Intapan P.M., Yamasaki H., Laummaunwai P., Sawanyawisuth K., Wongkham C., Tayapiwatana C., Kitkhuandee A., Lulitanond V., Nawa Y. (2013). A recombinant matrix metalloproteinase protein from *Gnathostoma spinigerum* for serodiagnosis of neurognathostomiasis. Korean J. Parasitol..

[36] Uparanukraw P., Morakote N., Harnnoi T., Dantrakool A. (2001). Molecular cloning of a gene encoding matrix metalloproteinase-like protein from *Gnathostoma spinigerum*. Parasitol. Res..

[37] Janwan P., Intapan P.M., Yamasaki H., Laummaunwai P., Sawanyawisuth K., Wongkham C., Tayapiwatana C., Kitkhuandee A., Lulitanond V., Nawa Y. (2013). Application of recombinatnt *Gnathostoma spinigerum* matrix mettaloproteinase-like protein for serodiagnosis of human gnathostomiasis by immunoblotting. Am. J. Trop. Hyg..

[38] Kongkerd N., Uparanukraw P., Morakote N., Sajid M., McKerrow J.H. (2008). Identification and characterization of a cathepsin L-like cysteine protease from Gnathostoma spinigerum. Mol. Biochem. Parasitol..

[39] Stachyra A., Zawistowska-Deniziak A., Basałaj K., Grzelak S., Gondek M., Bień-Kalinowska J. (2019). The Immunological Properties of Recombinant Multi-Cystatin-Like Domain Protein From *Trichinella Britovi* Produced in Yeast. Front. Immunol..

[40] Nuamtanong S., Reamtong O., Phuphisut O., Chotsiri P., Malaithong P., Dekumyoy P., Adisakwattana P. (2019). Transcriptome and excretory-sectroy proteome of infective-stage larvae of the nematode *Gnathostoma spinigerum* reveal potential immunodiagnostic targets for development. Parasite.

[41] Aebersold R., Mann M. (2003). Mass spectrometry-based proteomics. Nature.

[42] Barrett J., Jefferies J.R., Brophy P.M. (2000). Parasite proteomics. Parasitol. Today.

[43] Mutapi F. (2012). Helminth parasite proteomics: From experimental models to human infections. Parasitology.

[44] Chen W., Wang X., Li X., Lv X., Zhou C., Deng C., Lei H., Men J., Fan Y., Liang C. (2014). Molecular characterization of cathepsin B from *Clonorchis sinensis* excretory/secretory products and assessment of its potential for serodiagnosis of clonorchiasis. Parasit Vectors.

[45] Sun G.G., Wang Z.O., Liu C.Y., Jiang P., Liu R.D., Wen H., Qi X., Wang L., Cui J. (2015). Early serodiangosis of trichinellosis by ELISA using excretory-secretory antigens of *Trichinella spiralis* adult worms. Parasit Vectors.

[46] Ranganathan S., Garg G. (2009). Secretome: Clues into pathogen infection and clinical applications. Genome Med..

[47] Vibanco-Pérez N., Durán-Avelar M.D.J., Zambrano-Zaragoza J.F., Ventura-Ramón G.H. (2015). Proteases secreted by Gnathostoma binucleatum degrade fibronectin and antibodies from mammals. Helminthologia.

[48] Sorsa T., Tervahartiala T., Leppilahti J., Hernandez M., Gamonal J., Tuomainen M.A., Lauhio A., Pussinen P.J., Mäntylä P. (2011). Collagenase-2 (MMP-8) as a point-of-care biomarker in periodontitis and cardiovascular diseases. Therapeutic response to non-antimicrobial properties of tetracyclines. Pharmacol. Res..

[49] Law R.H.P., Zhang Q., McGowan S., Buckle A.M., A Silverman G., Wong W., Rosado C.J., Langendorf C.G., Pike R.N., I Bird P. (2006). An overview of the serpin superfamily. Genome Biol..

[50] Song Y.Y., Zhang Y., Ren H.N., Sun G.G., Qi X., Yang F., Jiang P., Zhang X., Cui J., Wang Z.Q. (2018). Characterization of a serine protease inhibitor from Trichinella spiralis and its participation in larval invasion of host’s intestinal epithelial cells. Parasites Vectors.

[51] Wongkamchai S., Chiangjong W., Sinchaikul S., Chen S.-T., Choochote W., Thongboonkerd V. (2011). Identification of Brugia malayi immunogens by an immunoproteomics approach. J. Proteom..

[52] Campista-León S., Delgado-Vargas F., Ríos-Sicairos J., Landa A., Willms K., Bojórquez-Contreras N., Diaz-Camacho S.P., López-Moreno H.S., Mendoza-Hernández G. (2012). Identification of Immunodominant Peptides from Gnathostoma binucleatum. Am. J. Trop. Med. Hyg..

[53] Laummaunwai P., Intapan P.M., Wongkham C., Lulitanond V., Maleewong W. (2008). Identification of antigenic components of *Gnathostoma spinigerum* advanced third stage larvae by two-dimensional gel electrophoresis and mass spectrometry. Southeast Asian J. Trop. Med. Public Health.

[54] Janwan P., Intapan P.M., Laummaunwai P., Rodpai R., Wongkham C., Insawang T., Thanchomnang T., Sanpool O., Maleewong W. (2015). Proteomic analysis identification of antigenic proteins in *Gnathostoma spinigerum* larvae. Exp. Parasitol..

[55] Sugaroon S., Wiwanitkit V. (2003). *Gnathostoma* infective stage larvae in swamp eels (Fluta alba) at a metropolitan market in Bangkok, Thailand. Ann. Clin. Lab. Sci..

[56] Sieu T., Dung T., Nga N., Hien T., Dalsgaard A., Waikagul J., Murrell D. (2009). Prevalence of *Gnathostoma spinigerum* infection in wild and cultured swamp eels in Vietnam. J. Parasitol..

[57] Veríssimo C.M., Graeff-Teixeira C., Jones M.K., Morassutti A.L. (2019). Glycans in the roles of parasitological diagnosis and host-parasite interplay. Parasitology.

[58] Singh A. (2021). Glycoproteomics. Nat. Methods.

[59] Tissot B., North S.J., Ceroni A., Pang P., Panico M., Rosati M., Capone A., Haslam S.M., Dell A., Morris H.R. (2009). Glycoproteomics: Past, present and future. FEBS Lett..

[60] Mitcheson D.F., Tobin A.B., Alam M.M. (2015). Applying chemical genetic tools to the study of phospho-signalling pathways in malaria parasites. Biochim. Biophys. Acta.

